# Feasibility and Accuracy of a Fully Automated Right Ventricular Quantification Software With Three-Dimensional Echocardiography: Comparison With Cardiac Magnetic Resonance

**DOI:** 10.3389/fcvm.2021.732893

**Published:** 2021-10-21

**Authors:** Ashfaq Ahmad, He Li, Xiaojing Wan, Yi Zhong, Yanting Zhang, Juanjuan Liu, Ying Gao, Mingzhu Qian, Yixia Lin, Luyang Yi, Li Zhang, Yuman Li, Mingxing Xie

**Affiliations:** ^1^Department of Ultrasound Medicine, Union Hospital, Tongji Medical College, Huazhong University of Science and Technology, Wuhan, China; ^2^Clinical Research Center for Medical Imaging in Hubei Province, Wuhan, China; ^3^Hubei Province Key Laboratory of Molecular Imaging, Wuhan, China; ^4^Department of Ultrasound, The First Affiliated Hospital of SooChow University, Suzhou, China; ^5^Shenzhen Huazhong University of Science and Technology Research Institute, Shenzhen, China; ^6^Tongji Medical College and Wuhan National Laboratory for Optoelectronics, Huazhong University of Science and Technology, Wuhan, China

**Keywords:** right ventricle, right ventricular volume, right ventricular ejection fraction, three-dimensional echocardiography, cardiac magnetic resonance

## Abstract

**Background:** A novel, fully automated right ventricular (RV) software for three-dimensional quantification of RV volumes and function was developed. The direct comparison of the software performance with cardiac magnetic resonance (CMR) was limited. Therefore, the aim of this study was to test the feasibility, accuracy, and reproducibility of a fully automated RV quantification software against CMR imaging as a reference.

**Methods:** A total of 170 patients who underwent both CMR and three-dimensional echocardiography were enrolled. RV end-diastolic volume (RVEDV), RV end-systolic volume (RVESV), and RV ejection fraction (RVEF) were obtained using fully automated three-dimensional RV quantification software and compared with a CMR reference. For inter-technical agreement, Spearman correlation and Bland–Altman analysis were used.

**Results:** The fully automated RV quantification software was feasible in 149 patients. RVEDV and RVESV were underestimated, and RVEF was overestimated compared with CMR values. RV measurements obtained from the manual editing method correlated better with CMR values than that without manual editing (RVEDV, 0.924 vs. 0.794: RVESV, 0.955 vs. 0.854; RVEF, 0.941 vs. 0.781 respectively, all *p* < 0.0001) with less bias and narrower limit of agreement (LOA). The bias and LOA for RV volumes and EF using the automated software without and with manual editing were greater in patients with severely impaired RV function or low frame rate than those with normal and mild impaired RV function, or high frame rate. The fully automated RV three-dimensional measurements were highly reproducible.

**Conclusion:** The novel fully automated RV software shows good feasibility and reproducibility, and the measurements had a high correlation with CMR values. These findings support the routine application of the novel 3D automated RV software in clinical practice.

## Introduction

Right ventricular (RV) function has been demonstrated to be independently associated with poor clinical outcomes in patients with a variety of cardiac and pulmonary pathologies (1, 2). Although cardiac magnetic resonance (CMR) is considered to be the gold standard method for the quantification of RV volumes and function (3–5), it is not feasible for those patients with common contraindication of implanted magnetic device (left ventricular assist device) other than with non-MRI conditional devices like ferromagnetic material such as shrapnel, medical devices, claustrophobia, etc. Based on its availability, versatility, and lower cost, echocardiography is the first-line option for cardiac quantification. Given the complex anatomy of the right ventricle, three-dimensional echocardiography (3DE) provides more accurate RV function assessment than two-dimensional echocardiography (2DE) due to avoidance of geometric assumptions and foreshortened views (6). Numerous studies have demonstrated the accuracy of 3DE for RV measurements in comparison with CMR reference (7–10) and have also confirmed its incremental prognostic value over conventional 2DE indices (11–13). Despite these aforementioned advantages, however, widespread application of 3DE for RV assessments has not been incorporated into the clinical practice. This may be owing to it being time consuming and requiring training to obtain accurate 3DE for RV measurements.

Recently, a new fully automated 3D RV quantification software using machine learning algorithms (MLA) was introduced. Limited studies had validated its accuracy against CMR imaging (14, 15). However, the feasibility and accuracy of a fully automated 3D RV software in large subjects with various RV function has not been well established, and the effects of RV ejection fraction (RVEF) and frame rate (FR) on the accuracy of this novel software has not been investigated.

Therefore, the purposes of this study were (i) to validate the feasibility, accuracy, and reproducibility of the fully automated 3D software for RV volumes and function quantification against CMR imaging in a large population with a wide range of RV function; (ii) to determine the impact of severity of RVEF and FR on the accuracy of the fully automated 3D RV measurements.

## Methods

### Population and Study Design

A total of 170 patients who had undergone both CMR and 3DE examinations with a median of 1 day [interquartile range (IQR); 0–6) were prospectively enrolled in our study from March 2017 to June 2020. The patients with arrhythmia or poor image quality of 3DE were excluded. Twenty-one patients were excluded, and a total of 149 patients were included in our final analysis.

The patients were divided into four subgroups on the basis of CMR-derived RVEF to normal RV function, mildly impaired, moderately impaired, and severely impaired RV function (RVEF: normal above 51% in males and 52% in females, mildly impaired 41–51%, moderately impaired 31–40%, and severely impaired ≤ 30%) (16). According to the RV function assessed by 3DE, patients were divided into three subgroups (normal RV function, RVEF > 45%; mild to moderately impaired RV function, 30% < RVEF ≤ 45%; and severely impaired RV function, ≤ 30%) (17). Additionally, the entire study population were divided into two subgroups based on the median FR of 3DE ( ≤ 23 frames/s or >23 frames/s). The study protocol was approved by the institutional ethics board of Union Hospital, Tongji Medical College, Huazhong University of Science and Technology. All patients provided informed consents.

### Echocardiography

The RV-focused 2DE and 3DE data set were obtained by using a Philip echocardiographic system (EPIQ 7C, X5-1 and S5-1 probes, Philips Healthcare Andover, MA, USA). All subjects were told to lie on the left semirecumbent position with electrocardiogram (ECG) leads attached properly. 2DE images were obtained in the RV-focused apical four-chamber (A4C) view and ensured to avoid the foreshortening of the right ventricle. In the RV-focused A4C view in full volume mode, 3DE images were obtained with a median FR of 23 (IQR [20-32]) frames/s, for four cardiac cycles with short breath hold. The acquisition quality would be considered inappropriate if endocardial boundary in one segment was not clear in the A4C view. 2D and 3D images were stored for offline study. The stored data set were transferred to a workstation where they were studied and analyzed for RV quantification with the fully automated software. The RV-focused A4C views with clear endocardial border were selected. Five landmarks were ensured in the apical, coronal, and basal short-axis views.

### Three-Dimensional Echocardiography Auto Right Ventricular Analysis

**Figure 1:**
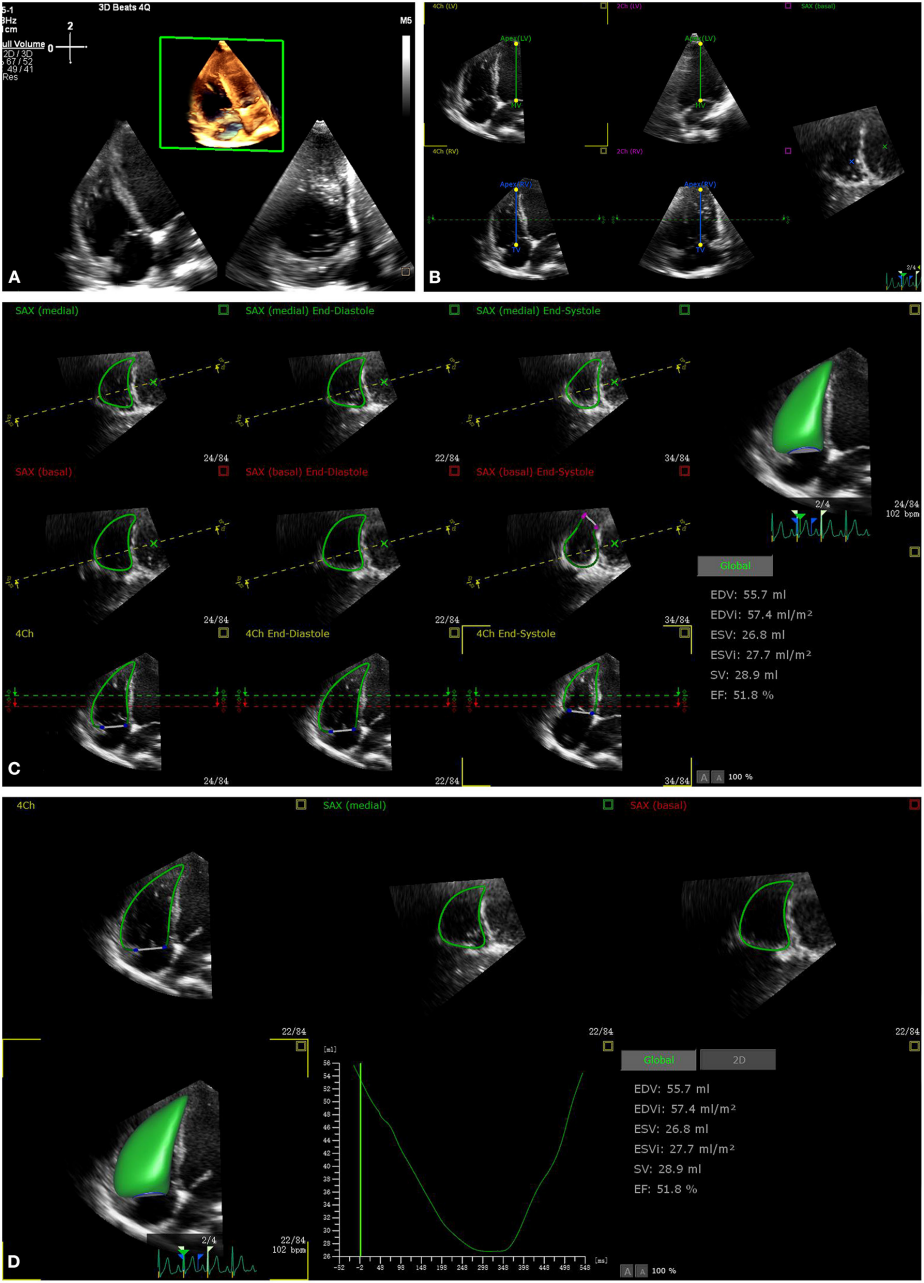
The process of fully automated right ventricular (RV) analysis. After retrieving the RV focus three-dimensional echocardiography (3DE) data aiming for RV analysis **(A)**, start the software, automatically adjust the five landmarks **(B)**, automatically determine the RV border at end diastole and end systole **(C)**, and provide the results within 15 s **(D)**.

The acquisition of a 3DE data set was subjectively assessed to ensure the complete visualization of the endocardial border in 3DE acquisition in three views (A4C, coronal, and basal short-axis views) (Figure 1). The image quality of the 3D data set was judged subjectively considering the signal-to-noise ratio and complete visualization of the RV endocardium, and was categorized on a scale from 1 to 4 (from poor to excellent) (10). The image quality was considered as poor if ultrasound dropout was present in the coronal view more than one half of RV free wall (18). RV full volume 3DE data sets were analyzed by a novel fully automated 3D RV quantification software (3D auto RV, Philips Healthcare). The software detected the RV endocardial border using artificial intelligence, which consists of initial RV orientation and global shape recognition, and then 3D endocardial speckle tracking was conducted throughout one cardiac cycle. Once a good image frame was focused, the automated software was started by one click to adjust a 3D endocardial tracking cast of the RV at end diastole, and 3D speckle tracking analysis was subsequently performed. Finally, the software automatically generated the RV volume–time curves, from which the RV end-diastolic volume (RVEDV), RV end-systolic volume (RVESV), and RVEF were obtained.

When the requested contour adjustment was performed on 2D images in both long- and short-axis views at end diastole, a new speckle tracking dataset was obtained. If the software made an erroneous 3D RV cast, a revision analysis would be performed. The subject would be discarded if the new 3D cast was still erroneous.

Results (RVEDV, RVESV, and RVEF) with manual editing were also obtained through manual adjustment in the end-diastolic frame and end-systolic frame at both long- and short-axis views. These data (with or without manual editing) were compared with those from CMR for accuracy validation.

### Cardiac Magnetic Resonance Imaging

CMR imaging was performed using a 1.5T scanner (MAGNETOM Area, Siemens Healthcare, Germany) with a phased array of cardiovascular coil. For the identification of the long axis of the heart, retrospective ECG-gated localizing echo spine sequences were used. Steady-state free precession (SSFP) dynamic gradient-echo cine loop was acquired using retrospective ECG gating and parallel imaging technique with 6-mm slice thickness of the imaging plane. The parameters were set as 340 × 255-mm field of view, 256 × 205 scan matrix, and 80° flip angle. The median FR was 32 [IQR (28 to 36)] frames/s with 10- to 15-s breath holds.

### Cardiac Magnetic Resonance Analysis

CMR imaging was analyzed with conventional CMR software (Argus Siemens Medical Solution) by a sophisticated technician who was blinded to echocardiographic findings. Disc summation method of multiple short-axis views was used for the calculation of RVEDV and RVESV. RVEF was calculated through the standard formula (RVEF = RVEDV – RVESV/RVEDV ^*^ 100%).

### Reproducibility

Intraobserver, interobserver, and test–retest variability was used to assess the reproducibility of the fully automated software on RV measurements. For intraobserver variability, 20 patients were selected randomly, and the analysis was performed again 2 weeks later by the same observer, while for interobserver variability, the same 20 patients were examined 2 weeks later by another observer who was blinded to the initial results. Test–retest variability was assessed by two different observers acquiring and analyzing the data independently at different time points. Intraobserver, interobserver, and test–retest variability was evaluated by coefficients of variation (CoV), quantified as the absolute differences between the repeated two measurements in percentages of their mean, and intraclass correlation coefficient (ICC).

### Analysis Time

The analysis time for the software in both modes (fully automated without and with manual editing) was measured in 20 patients. For fully automated mode, the time was measured from clicking the analysis button to displaying the results. While for manual editing mode, the analysis time also included tracking revision and endocardial border adjustment in both end-diastolic and end-systolic frames.

### Statistical Analysis

Continuous variables are presented as mean ± standard deviation (SD), or median and IQR, while categorical variables are presented as frequency (percentage). Shapiro–Wilk test was used to determine whether the data conforms to the normal distribution. Since these data were not normally distributed, the Friedman's analysis with Wilcoxon comparison was used to compare the results among the three groups. Spearman correlation analysis was used to assess the correlation of between variables. Bland–Altman analysis was used for the determination of bias and limit of agreement (LOA). For the agreement of categorization between 3DE with fully automated analysis and CMR, and between 3DE auto edit analysis and CMR, the weighted Kappa statistical analysis was used. Correlation coefficient was compared using MedCalc version 19.0.4 (MedCalc Software, Ostend, Belgium) for the RV measurements obtained from the automated software without or with manual editing against CMR imaging as a reference. Data were analyzed using SPSS version 22 (SPSS Inc., Chicago, IL, USA) and GraphPad prism 8.0 (GraphPad Software, San Diego, CA, USA). A two-sided *p* < 0.05 was considered to be statistically significant.

## Results

### Study Population

The fully automated RV quantification software was feasible in 149 (87.6%) subjects. Clinical characteristics of study subjects are shown in Table 1. Image quality by 3D data set was graded excellent in 18.1% (*n* = 27), good in 50.3% (*n* = 75), fair in 23.5 % (*n* = 35), and poor in 8.1% (*n* = 12). The mean age of the subjects was 46 ± 15 years, and 115 (77.18%) were men. The entire population consisted of 12 (8.05%) healthy subjects and 137 (91.95%) patients. Among the 137 patients, 19 patients were diagnosed with ischemic heart disease, 53 with dilated cardiomyopathy, 18 with heart transplantation recipients, 15 with valvular heart disease, 16 with hypertrophic cardiomyopathy, eight with hypertensive heart disease, three with multiple myeloma, two with perinatal cardiomyopathy and one with uremic cardiomyopathy, one with rheumatic heart disease, and one with viral myocarditis. Forty-eight percent of the patients [dilated cardiomyopathy (*n* = 52) and ischemic heart disease (*n*=19)] with end-stage heart failure were included in the waiting list of heart transplantation.

**Table 1:** Clinical characteristics of study population (*n* = 149).

Age (years)	46 ± 15
Gender (male/female)	115/34
Height (cm)	165 ± 21
Weight (kg)	67 ± 15
Body surface area (m^2^)	1.75 ± 0.23
Systolic blood pressure (mmHg)	118 ± 20
Diastolic blood pressure (mmHg)	76 ± 15
Heart rate at 3DE (bpm)	86 ± 14
Heart rate at CMR (bpm)	85 ± 15
Clinical Diagnose	
Normal	12 (8.05%)
Ischemic heart disease	19 (12.75%)
Dilated cardiomyopathy	53 (35.57%)
Valvular heart disease	15 (10.07%)
Hypertrophic cardiomyopathy	16 (10.74%)
Heart transplantation recipients	18 (12.08%)
Hypertensive heart disease	8 (5.37%)
Multiple myeloma	3 (2.01%)
Perinatal cardiomyopathy	2 (1.34%)
Uremic cardiomyopathy	1 (0.67%)
Rheumatic heart disease	1 (0.67%)
Viral myocarditis	1 (0.67%)

*Data are expressed as n (%), mean ± SD*.

### Right Ventricular Volumes and Ejection Fraction Analysis

**Figure 2:**
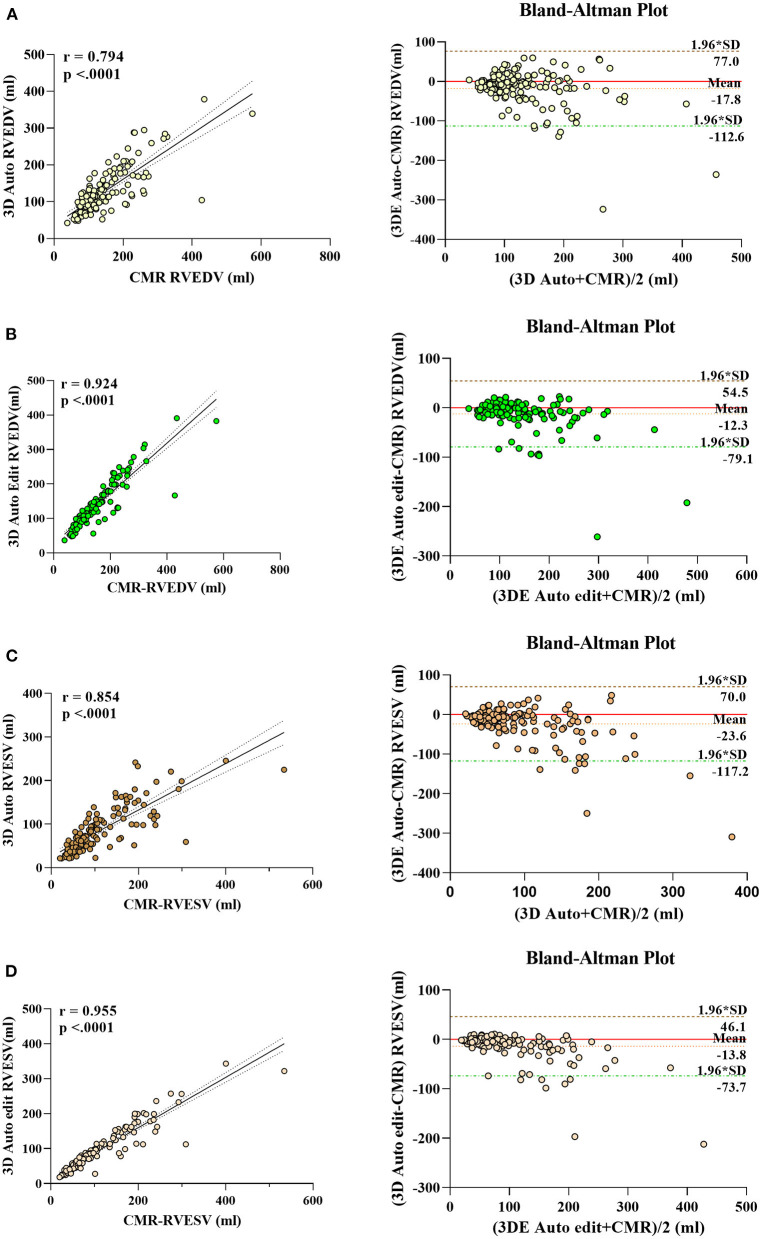
Linear correlation and Bland–Altman analysis of RV volumes with 3D auto RV and edit method, with CMR. RVEDV auto **(A)**, RVEDV edit **(B)**, RVESV auto **(C)**, and RVESV edit **(D)**.

**Figure 3:**
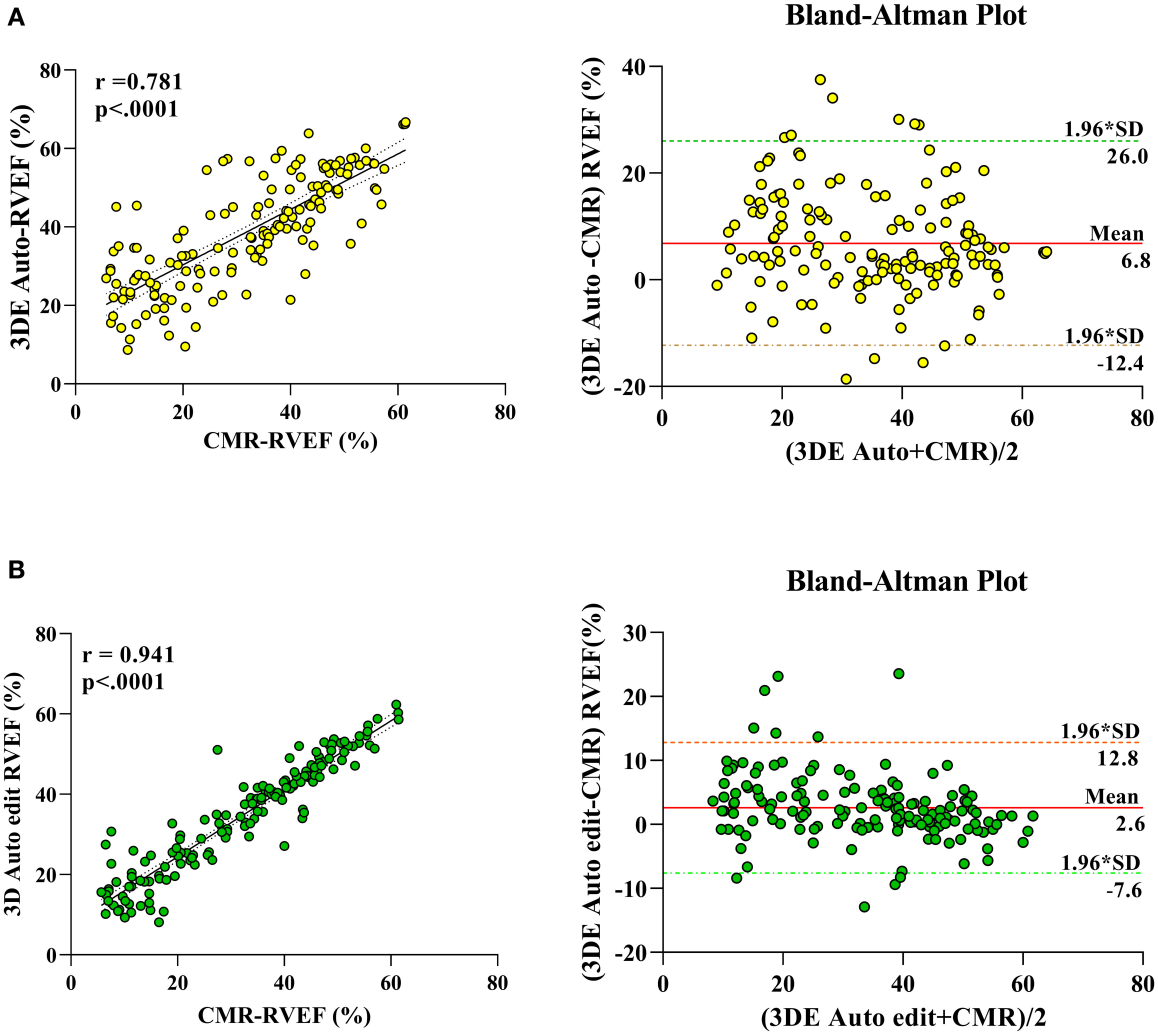
Linear correlation and Bland–Altman analysis of RVEF with 3D auto RV and edit method, with CMR. 3DE auto EF with CMR **(A)**, 3D auto edit EF with CMR **(B)**.

**Figure 4:**
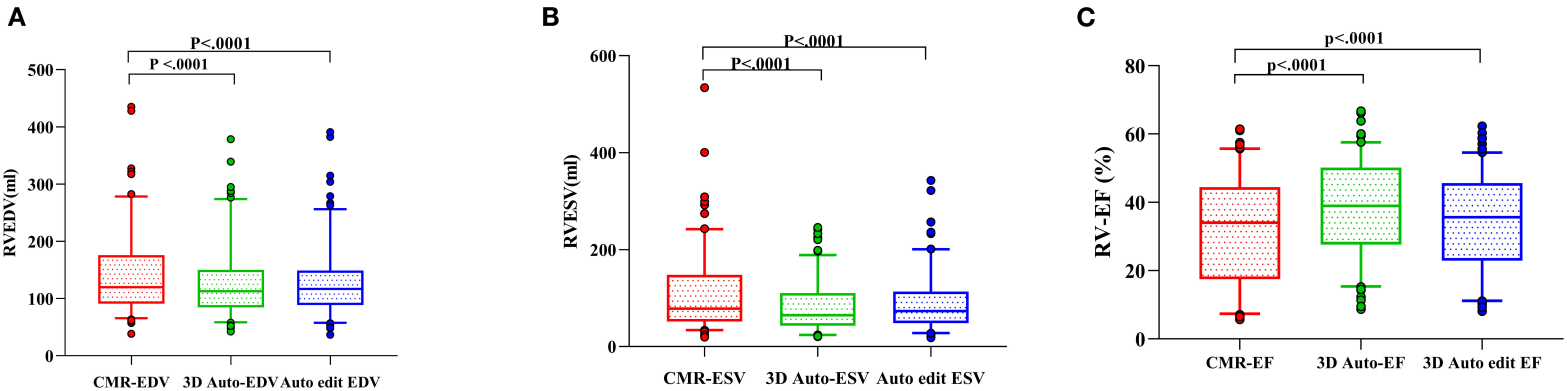
RV measurements comparison among three methods [3D auto RV, manual editing method, and cardiac magnetic resonance (CMR)]. Right ventricular end-diastolic volume (RVEDV) **(A)**, right ventricular end-systolic volume (RVESV) **(B)**, and right ventricular ejection fraction (RVEF) **(C)**.

The correlation and Bland–Altman analysis for RV volumes and EF using 3D auto RV software with/without manual editing against CMR as a reference are presented in Table 2 and Figures 2, 3. Comparison of RV measurements among three methods are shown in Figure 4. The 3D Auto RV quantification software overestimated the RVEF and slightly underestimated RV volumes in comparison with CMR [RVEF: 38.9 (27.6–50.1)% vs. 34.0 (17.5–44.5)%, RVEDV: 112.9 (84.6–150.0) ml vs. 119.8 (91.1–175.8) ml; RVESV: 64.7 (42.9–110.3) ml vs. 78.1 (51.7–147.7) ml, all *p* < 0.0001]. The results were improved with the manual editing method and successfully approximated the RV volumes and RVEF against CMR [RVEF: 35.6 (22.9–45.6)% vs. 34.0 (17.5–44.5)%; RVEDV: 116.9 (88.6–148.9) ml vs. 119.8 (91.1–175.8) ml; RVESV: 73.6 (48.1–113.7) ml vs. 78.1 (51.7–147.7), all *p* < 0.0001). Strong correlations were observed between RV measurements obtained from 3D Auto RV and CMR (RVDEV: r = 0.794; RVESV: r = 0.854; RVEF: r = 0.781; all *p* < 0.0001). The correlations further improved when we performed manual editing method (RVEDV: r = 0.924; RVESV: r = 0.955; RVEF: r = 0.941, all *p* < 0.0001) in comparison with CMR. RV measurements obtained from the manual editing method correlated better with CMR values than that without manual editing (RVEDV, 0.924 vs. 0.794: RVESV, 0.955 vs. 0.854; RVEF, 0.941 vs. 0.781, respectively, all *p* < 0.0001). In addition, RVEF using 3D auto RV software with manual editing had smaller biases and narrower LOAs against CMR values than that of 3D auto RV quantification (bias: 2.6 and 6.8, LOA: 10.2 and 19.2, respectively). A similar trend was also observed in RVEDV and RVESV.

**Table 2:** Comparison between cardiac magnetic resonance (CMR) and three-dimensional (3D) auto right ventricular (RV) quantification with or without manual editing.

**Variable**	**Novel software (*n* = 149)**	**CMR (*n* = 149)**	**Correlation**	***p*-Value**	**Bias**	**LOA**
**RVEF (%)**
3D auto RV	38.9 (27.6–50.1)	34.0 (17.5–44.5)	r = 0.781	<0.0001	6.8	19.2 (−12.4 to 26.0)
3D auto edit	35.6 (22.9–45.6)	34.0 (17.5–44.5)	r = 0.941	<0.0001	2.6	10.2 (−7.6 to 12.8)
**RVEDV (ml)**
3D auto RV	112.9 (84.6–150.0)	119.8 (91.1–175.8)	r = 0.794	<0.0001	−17.8	94.8 (−112.6 to 77.0)
3D auto edit	116.9 (88.6–148.9)	119.8 (91.1–175.8)	r = 0.924	<0.0001	−12.3	66.8 (−79.1 to 54.5)
**RVESV (ml)**
3D auto RV	64.7 (42.9–110.3)	78.1 (51.7–147.7)	r = 0.854	<0.0001	−23.6	93.6 (−117.2 to 70.0)
3D auto edit	73.6 (48.1–113.7)	78.1 (51.7–147.7)	r = 0.955	<0.0001	−13.8	59.9 (−73.7 to 46.1)

*Data are expressed as median and interquartile range. LOA, limit of agreement*.

The effects of RV function on RV volumes and EF using 3D auto RV software with/without manual editing compared with CMR measurements are summarized in Table 3. For RV volumes and RVEF, biases and LOA with or without manual editing were larger in patients with severely impaired RV function compared with those with normal or mild impaired RV function. Likewise, RV volumes and RVEF obtained by the manual editing method had smaller biases and narrower LOA compared with 3D auto RV software without manual editing measurements in patients with normal or mildly, moderately, and severely impaired RV function.

**Table 3:** The effect of right ventricular ejection fraction (RVEF) on 3D-Auto RV quantification with or without manual editing compared with CMR.

**Variable**	**Novel software (*n* = 149)**	**CMR (*n* = 149)**	**Correlation**	***p*-Value**	**Bias**	**LOA**
**NORMAL RV FUNCTION (*****n*** **=** **16)**						
**RVEF (%)**					
3D auto RV	57.15 (55.82–59.49)	55.52 (52.99–57.37)	r = 0.835	0.0001	2.1	7.3 (−5.2 to 9.4)
3D auto edit	56.17 (53.63–58.25)	55.52 (52.99–57.37)	r = 0.911	<0.0001	0.8	3.1 (−2.3 to 3.9)
**RVEDV (ml)**					
3D auto RV	74.04 (66.31–88.31)	78.87 (73.35–92.47)	r = 0.782	0.0006	−5.8	22.3 (−28.1 to 16.5)
3D auto edit	77.93 (71.38–81.81)	78.87 (73.35–92.47)	r = 0.905	<0.0001	−5.4	13.1 (−18.5 to 7.7)
**RVESV (ml)**					
3D auto RV	34.44 (28.94–38.51)	36.95 (32.84–42.23)	r = 0.732	0.001	−4.2	9.8 (−14.0 to 5.6)
3D auto edit	35.27 (31.69–40.00)	36.95 (32.84–42.23)	r = 0.841	<0.0001	−3.2	9.0 (−12.2 to 5.8)
**MILDLY IMPAIRED RV FUNCTION (*****n*** **=** **35)**						
**RVEF (%)**					
3D auto RV	48.96 (45.62–52.70)	45.70 (43.40–47.50)	r = 0.576	0.0003	2.8	9.1 (−6.3 to 11.9)
3D auto edit	47.28 (44.28–49.62)	45.70 (43.40–47.50)	r = 0.655	<0.0001	1.1	7.6 (−6.5 to 8.7)
**RVEDV (ml)**					
3D auto RV	87.90 (68.80–114.20)	105.5 (84.20–116.8)	r = 0.774	<0.0001	−7.6	40.3 (−47.9 to 32.7)
3D auto edit	96.20 (76.80–115.50)	105.5 (84.20–116.8)	r = 0.873	<0.0001	−6.1	20.9 (−27.0 to 14.8)
**RVESV (ml)**					
3D auto RV	44.32 (36.50–58.60)	56.40 (43.40–65.96)	r = 0.726	<0.0001	−7.0	32.2 (−39.2 to 25.2)
3D auto edit	50.98 (42.56–60.52)	56.40 (43.40–65.96)	r = 0.782	<0.0001	−3.4	22.1 (−25.5 to 18.7)
**MODERATELY IMPAIRED RV FUNCTION (*****n*** **=** **32)**						
**RVEF (%)**					
3D auto RV	39.80 (36.07–45.81)	35.85 (33.78–38.70)	r = 0.407	0.020	5.1	17.0 (−11.9 to 22.1)
3D auto edit	38.54 (35.62–41.40)	35.85 (33.78–38.70)	r = 0.499	0.003	1.9	8.3 (−6.4 to 10.2)
**RVEDV (ml)**					
3D auto RV	103.0 (76.67–117.6)	108.1 (84.10–132.6)	r = 0.604	0.0002	−8.6	45.5 (−54.1 to 36.9)
3D auto edit	107.9 (86.25–122.3)	108.1 (84.10–132.6)	r = 0.853	<0.0001	−8.5	33.4 (−41.9 to 24.9)
**RVESV (ml)**					
3D auto RV	64.72 (42.63–81.10)	68.75 (52.10–88.25)	r = 0.595	0.0003	−8.0	42.9 (−50.9 to 34.9)
3D auto edit	65.28 (45.56–78.62)	68.75 (52.10–88.25)	r = 0.772	<0.0001	−7.8	29.2 (−37.0 to 21.4)
**SEVERELY IMPAIRED RV FUNCTION (*****n*** **=** **66)**						
**RVEF (%)**					
3D auto RV	27.06 (21.50–33.17)	15.75 (10.20–22.48)	r = 0.312	0.010	11.3	20.2 (−8.9 to 31.5)
3D auto edit	24.53 (16.86–29.96)	15.75 (10.20–22.48)	r = 0.463	<0.0001	7.9	16.5 (−8.6 to 24.4)
**RVEDV (ml)**					
3D auto RV	148.2 (114.3–196.5)	181.0 (130.5–229.0)	r = 0.594	<0.0001	−29.4	133.5 (−162.9 to 104.1)
3D auto edit	148.8 (123.1–204.1)	181.0 (130.5–229.0)	r = 0.826	<0.0001	−21.3	96.7 (−118.0 to 75.4)
**RVESV (ml)**					
3D auto RV	116.8 (82.70–150.8)	157.8 (101.1–198.8)	r = 0.609	<0.0001	−43.8	130.9 (−174.7 to 87.1)
3D auto edit	119.7 (89.71–163.4)	157.8 (101.1–198.8)	r = 0.862	<0.0001	−27.5	85.3 (−112.8 to 57.8)

*Data are expressed as median and interquartile range, LOA, limit of agreement; RVEDV, right ventricular end-diastolic volume; RVESV, right ventricular end-systolic volume*.

The agreement of categorization (RVEF <30%, RVEF ≥ 30% but RVEF <45%, and RVEF ≥ 45%) between 3DE with fully automated analysis and CMR, and 3DE with manual edit method and CMR, are presented in Table 4. We found that the agreement of categorization between 3DE with manually edited analysis and CMR (kappa value, 0.866, 95% CI: 0.819 to 0.914, p <0.0001) was better than that of between 3DE with fully automated analysis and CMR (kappa value, 0.674, 95% CI: 0.603 to 0.745, *p* < 0.0001).

**Table 4:** The agreement of categorization between 3D auto RV with/without manual edit and CMR.

**CMR and 3DE Auto**
**Kappa** **=** **0.674 (0.603 to 0.745) 95% CI**		**CMR**	
**3DE auto**	RVEF <30%	RVEF ≥30% but RVEF <45%	RVEF ≥45%
RVEF <30%	44	16	6
RVEF ≥30% but RVEF <45%	3	27	17
RVEF ≥45%	0	2	34
**CMR and 3DE auto edit**
**Kappa** **=** **0.866 (0.819 to 0.914) 95% CI**		**CMR**	
**3DE auto edit**	RVEF <30%	RVEF ≥30% but RVEF <45%	RVEF ≥45%
RVEF <30%	56	1	0
RVEF ≥30% but RVEF <45%	9	42	3
RVEF ≥45%	1	4	33

*CI, confidence interval*.

When the effect of FR was evaluated, there was no significant impact on the RVEF. However, the biases and LOA for RV volumes were larger in patients with FR ≤ 23 frame/s regardless of whether the manual editing was performed. Additionally, the biases and LOA for RV volumes and RVEF derived from the manual editing method was smaller than those without manual editing irrespective of FR (Table 5).

**Table 5:** The effects of frame rate (FR) on 3D auto RV quantification with/without manual editing compared with CMR.

**Variable**	**Novel Software (*n* = 149)**	**CMR (*n* = 149)**	**Correlation**	***p*-Value**	**Bias**	**LOA**
**VOLUME RATE** **≤23 FRAME/S (*****n*** **=** **85)**						
**RVEF%**						
3D auto RV	35.7 (26.6–48.9)	31.8 (14.7–44.8)	r = 0.753	<0.0001	7.1	20.7 (−13.6 to 27.8)
3D auto edit	32.7 (19.6–45.6)	31.8 (14.7–44.8)	r = 0.938	<0.0001	3.0	10.5 (−7.5 to 13.5)
**RVEDV (ml)**
3D auto RV	104.5 (79.3–167.8)	121.7 (93.8–187.0)	r = 0.824	<0.0001	−21.4	105.2 (−126.6 to 83.8)
3D auto edit	115.5 (90.5–179.0)	121.7 (93.8–187.0)	r = 0.933	<0.0001	−14.3	75.5 (−89.8 to 61.2)
**RVESV (ml)**	
3D auto RV	64.3 (42.3–121.4)	78.1 (55.1–165.1)	r = 0.867	<0.0001	−28.4	109.5 (−137.9 to 81.1)
3D auto edit	73.6 (50.8–146.5)	78.1 (55.1–165.1)	r = 0.967	<0.0001	−14.5	68.7 (−83.2 to 54.2)
**VOLUME RATE** **>23 FRAME/S (*****n*** **=** **64)**						
**RVEF%**						
3D auto RV	41.7 (28.6–54.7)	34.9 (24.5–44.4)	r = 0.754	<0.0001	6.2	18.0 (−11.8 to 24.2)
3D auto edit	38.7 (25.0–46.1)	34.9 (24.5–44.4)	r = 0.921	<0.0001	2.0	9.8(−7.8 to 11.8)
**RVEDV (ml)**
3D auto RV	114.3 (88.2–145.2)	118.2 (82.6–170.3)	r = 0.753	<0.0001	−13.0	78.8 (−91.8 to 65.8)
3D auto edit	117.2 (79.8–146.5)	118.2 (82.6–170.3)	r = 0.900	<0.0001	−12.1	53.5(−65.6 to 41.4)
**RVESV (ml)**
3D auto RV	65.3 (43.4–99.4)	78.8 (49.8–129.1)	r = 0.837	<0.0001	−17.0	66.1 (−83.1 to 49.1)
3D auto edit	73.7 (46.8–102.4)	78.8 (49.8–129.1)	r = 0.931	<0.0001	−12.7	46.6 (−59.3 to 33.9)

*Data are expressed as median and interquartile range. LOA, limit of agreement*.

### Reproducibility

The intraobserver, interobserver, and test–retest variability of the 3D Auto RV software with and without manual editing method are shown in Table 6. The intraobserver and interobserver reproducibility of the fully automated method was excellent, as reflected by high ICC (intraobserver: 1; interobserver: 1). For 3D manual editing method, the intraobserver and interobserver reproducibility for all measurements was good, as reflected by low CoV and high ICC (CoV: 2.2–6.2%, ICC: 0.989–0.999). Test–retest variability of 3D fully automated method without or with manual editing method was low, as evidence by low CoV (fully automated method: 4.3–7.8%; manual editing method: 3.3–8.7%) and excellent ICC (fully automated method: 0.974–0.980; manual editing method: 0.943–0.982).

**Table 6:** Reproducibility of 3D auto RV measurements with/without manual editing and CMR for RV volumes and EF.

**Method**	**Variable**	**Intra-observer**		**Inter-observer**		**Test–retest**	
		**%Variability, Mean ± SD**	**ICC**	**%Variability, Mean ± SD**	**ICC**	**%Variability, Mean ± SD**	**ICC**
3D fully automated method	RVEDV (ml)	0	1	0	1	6.2 ± 7.1	0.974
	RVESV (ml)	0	1	0	1	7.8 ± 8.0	0.980
	RVEF (%)	0	1	0	1	4.3 ± 4.5	0.980
3D manual editing method	RVEDV (ml)	2.2 ± 1.7	0.999	2.7 ± 3.2	0.997	8.6 ± 5.4	0.943
	RVESV (ml)	4.1 ± 3.6	0.996	4.9 ± 4.2	0.995	8.7 ± 6.2	0.965
	RVEF (%)	5.7 ± 5.4	0.990	6.2 ± 6.7	0.989	3.3 ± 5.2	0.982
CMR	RVEDV (ml)	4.1 ± 1.8	0.989	5.5 ± 2.1	0.979	4.9 ± 1.9	0.983
	RVESV (ml)	7.5 ± 5.4	0.982	9.5 ± 4.6	0.971	9.3 ± 4.3	0.971
	RVEF (%)	8.8 ± 5.8	0.928	9.8 ± 5.6	0.908	10.8 ± 4.9	0.887

*ICC, intra-class correlation coefficient; CoV, coefficient of variation*.

### Time of Analysis

The median time of analysis for 3D fully automated RV quantification software was 12s (IQR 9–14 s), which is much shorter than that of the manual editing method (71s; IQR 62–76 s).

## Discussion

The main findings of the present study can be summarized as (i) RV volumes and function using fully automated RV quantification software were strongly correlated with CMR values. (ii) The manual editing method improved measurement accuracy. (iii) The bias and LOAs for the RV volumes and EF using the 3D auto RV software were smaller in patients with normal or mild impaired RV function or FR >23 frame/s than those with severely impaired RV function or FR ≤ 23 frame/s regardless of whether the manual editing was performed. (iv) The fully automated software showed excellent reproducibility and reduced the duration of analysis.

### Accuracy of the Fully Automated Right Ventricular Quantification Software With/Without Manual Editing Method Compared With Cardiac Magnetic Resonance

3D assessment is required for accurate analysis of the right ventricle due to its irregular shape and complex contract mode (6). Nowadays, 3DE is recommended for RV size and function assessment according to updated recommendation from the American Society of Echocardiography (ASE) as well as European Association of Cardiovascular Imaging (EACVI). (6) In 2010, a 3D RV quantification software was first introduced, and its accuracy was tested against CMR and multidetector CT (19). A subsequent study demonstrated that RV volumes obtained by this software were significantly underestimated in patients with congenital heart diseases or moderate to severe RV dilatation in comparison with CMR (20). Recently, a new and semi-automated 3D RV quantification software was developed, and it can provide excellent accuracy and reproducibility for the measurement of RV volumes and function compared with CMR (10). Nevertheless, the software still needs manual input, which may leads to higher variability.

In order to overcome the variability caused by manual input, a novel, fully automated RV quantification software using MLA has been developed and validated against CMR. It is reported that the fully automated software showed excellent accuracy and reproducibility compared with CMR. This new software without manual editing worked in one-third of the population. However, manual editing was still required in the rest of the study population (14). A recent study performed by Otani et al. demonstrated that a novel fully automated RV quantification software underestimated RV volumes in comparison with CMR. However, there was no significant difference in RVEF between the fully automated method and CMR (15). Most recently, another research demonstrated that RVEF using the fully automated software was associated with adverse clinical events (17).

However, these two studies were limited by the relatively small number of study subjects (14, 15); the fully automated 3D software was not fully validated against CMR, whereas our study focused on a large population with a wide range of RV sizes and function. Consistent with previous 3D RV studies (10, 11, 14, 15), our data showed that RV volumes by the novel 3D automated software strongly correlated with CMR measurements. Moreover, our results further indicated that the correlation became higher when we performed manual editing. Meanwhile, in our study, the fully automated software slightly underestimated the RV volumes, similar to the previous study (10, 11, 14, 15, 21). With manual editing, the biases for RV volumes were smaller than in previous reports (11, 21).

The prior results regarding the accuracy of novel 3D automated RV quantification software for RVEF measurement in comparison with CMR are discordant. Genovese et al. demonstrated that RVEF by the 3D automated software was underestimated against CMR imaging (14), whereas other studies showed no significant difference in RVEF between the fully automated method and CMR (15, 17). In the present study, our results showed that the 3D automated RV software overestimated RVEF compared with CMR in a large population, similar to previous reports (11, 21). The 3D automated RV software appeared to be excellent accuracy in quantifying RV volumes and EF using CMR as a reference, as reflected by small biases and narrow LOA. Advantages provided by the 3D automated RV software in terms of superior accuracy and usability could be useful particularly for those patients requiring a close follow-up of RV function that cannot be assessed by serial CMR imaging.

### Impact of Right Ventricular Ejection Fraction and Frame Rate on the Three-Dimensional Auto Right Ventricular Quantification Software in Comparison With Cardiac Magnetic Resonance

The degree of RVEF has a great impact on 3D auto RV measurements and is the major factor contributing to the difference in RV quantification against CMR. Our study demonstrated that the biases for RV volumes and EF using the 3D auto method were larger in patients with severely impaired RV function. The biases improved when manual editing was performed. These results are in keeping with the study of Tsang et al. which showed that the biases and LOA for LV volumes using the 3D auto LV quantification software were larger in patients with reduced LVEF (22). Previous studies reported that the extent of underestimation of RV volumes in 3DE was greater in the case of RV dilatation than that of normal RV size (9). As patients with severely impaired RV function usually present with an enlarged right ventricle and abundant RV trabeculae, the software tracks further inside due to incognizance of these trabeculae and RV free wall, thus, leading to greater underestimation of RV volumes by 3DE in such subjects.

Our study demonstrated that the 3D auto RV software performed worse in subjects with severely impaired RV function, as evidenced by larger bias and wider LOA. The accuracy become higher when we performed manual editing. Although the 3D auto RV software worked worse in patients with severely decreased RVEF, the 3DE with fully automated analysis also diagnosed that the patient had severely impaired RVEF regardless of whether the values were different.

The FR plays an important role in the accuracy of 3D auto RV measurements. Our study revealed that novel 3D auto analysis for RV volumes performed better in high FR (>23 frame/s) than those of low FR ( ≤ 23 frame/s) regardless of whether the manual editing was performed. These findings are similar to those in previous study, which demonstrated that the biases and LOA for LV volumes using the 3D auto LV quantification software were larger in patients with low volume rates (22). However, our study revealed that FR had no significant impact on the RVEF, which was consistent with the prior study regarding the effect of 3DE volume rate on the LVEF using the 3D auto LV quantification software (22).

In this study, we tested the performance of 3DE auto RV software against CMR for RV quantification. The impressive feature of this software is the more rapid analysis and excellent reproducibly. Nevertheless, our study revealed that 3D auto RV software performed worse in subjects with RV dilation. Moreover, the severe outlier may occur in patients with extremely dilated right ventricle. Therefore, the novel software is needed to learn a large amount of RV contour information with the diverse pathology-related geometric changes to enhance the accuracy in a pathology-dependent way. With future technological improvements and machine learning algorithmic refinements with additional training on larger data sets, the percentage of subjects with accurate fully automated RV measurements without manual edit will rise over time.

## Limitations

Although our study population consisted of a large number of patients with a wide range of RV sizes and function, it was still a single-center study with relatively limited sample size, which cannot be generalized to the whole community. Future multicenter studies with larger sample sizes are required to confirm our results. Forty-eight percent of the patients were included in the waiting list of heart transplantation; therefore, our study population cannot reflect the general population. Since both CMR and 3DE investigations were done at different times, there may be real volume discrepancies that limit agreement and trend. In addition, 3DE has lower temporal and spatial resolution than 2DE, which may have effects on RV measurements. Another important limitation is that the evaluated technology may depend on vendors regarding format and availability of images. Therefore, our findings might not be extrapolated to other vendors and software packages. The fully automated RV software may not be appropriate for patients with poor image quality. Hence, the accuracy of this software in individuals with poor image quality cannot be determined. Moreover, 3D full volume technique is strongly dependent on heart rate, so it is challenging in patients with arrhythmia such as atrial fibrillation. Thus, our results are not applicable to patients with irregular rhythms.

## Conclusion

The novel fully automated 3D software for RV volumes and EF quantification is highly feasible and reproducible. The values measured by novel fully automated RV software were strongly correlated with the CMR measurements in a large number of population with a diverse range of RV sizes and function. These findings support the routine use of the novel 3D auto RV software in daily clinical practice.

## Data Availability Statement

The raw data supporting the conclusions of this article will be made available by the authors, without undue reservation.

## Ethics Statement

The studies involving human participants were reviewed and approved by the Institutional Ethics Board of Union Hospital, Tongji Medical College, Huazhong University of Science and Technology. The patients/participants provided their written informed consent to participate in this study.

## Author Contributions

AA, YML, HL, XJW, and MXX: conception and design of the study. YTZ, YG, MZQ, and YXL: acquisition of data. YTZ, JJL, LXY, and LZ: analysis and interpretation of data. AA, YXL, and HL: drafting the article. AA, YML, HL, and MXX: revising the article. AA, YML, LZ, YG, and MXX: final approval of the article. All authors listed have made a substantial, direct and intellectual contribution to the work, and approved it for publication.

## Funding

This work was supported by the National Natural Science Foundation of China (Grant Nos. 81922033 and 81727805), the Fundamental Research Funds for the Central Universities (Grant No. 5003530082), the Key Research and Development Program of Hubei (Grant No. 2020DCD015), and the Shenzhen Science and Technology under Grant (SGDX20190917094601717).

## Conflict of Interest

The authors declare that the research was conducted in the absence of any commercial or financial relationships that could be construed as a potential conflict of interest.

## Publisher's Note

All claims expressed in this article are solely those of the authors and do not necessarily represent those of their affiliated organizations, or those of the publisher, the editors and the reviewers. Any product that may be evaluated in this article, or claim that may be made by its manufacturer, is not guaranteed or endorsed by the publisher.
